# Bladder Height-to-Width Ratio as a Predictor of Future Neurogenic Bladder Dysfunction in Patients With Anorectal Malformations

**DOI:** 10.7759/cureus.96326

**Published:** 2025-11-07

**Authors:** Shinsuke Yoshizawa, Kensuke Ohashi, Tetsuya Ishimaru, Hiroshi Kawashima

**Affiliations:** 1 Department of Pediatric Urology, Saitama Children's Medical Center, Saitama, JPN; 2 Division of Pediatric Surgery, National Center for Child Health and Development, Tokyo, JPN; 3 Department of Pediatric Surgery, Saitama Children's Medical Center, Saitama, JPN

**Keywords:** anorectal malformation, clean intermittent catheterization, height-to-width ratio, laparoscopic-assisted anorectoplasty, neurogenic bladder dysfunction

## Abstract

Introduction

This retrospective study evaluated the bladder height-to-width ratio (HWR) in neonates with anorectal malformations (ARMs) to assess its potential role as a predictor of neurogenic bladder dysfunction (NBD).

Objectives

This study aimed to evaluate whether HWR can serve as a predictor of future NBD in neonates with high and intermediate ARMs.

Study design

We retrospectively reviewed the medical records of 48 patients with high or intermediate ARMs who underwent laparoscopic-assisted anorectoplasty at our institution between January 2000 and December 2018. Maximum HWRs were measured using cystography performed shortly after birth. Patients were categorized into two groups: the NBD group (n = 8, requiring clean intermittent catheterization (CIC)) and the non-NBD group (n = 40, not requiring CIC). Additionally, odds ratios (ORs) were calculated for spinal abnormalities, sacral malformations, genetic anomalies, and ARM type.

Results

The NBD group included eight patients who required CIC, while the non-NBD group comprised 40 patients who did not. The median age of all patients was 11.56 years (7.8 years in the NBD group and 12.27 years in the non-NBD group). The NBD group had a significantly higher median HWR compared to the non-NBD group (1.942 vs. 1.432, p = 0.000029). An HWR cutoff of 1.842 yielded a sensitivity of 92.5% and a specificity of 75% for identifying patients who required CIC, with an area under the curve (AUC) of 0.888. Among the assessed variables, only sacral malformations showed a statistically significant association with NBD (OR 10.76, p = 0.0006).

Conclusion

Our findings suggest that neonates with high and intermediate ARMs already exhibit elevated HWR shortly after birth. HWR may thus serve as an early screening tool for predicting NBD in this population. An HWR threshold of 1.842 was identified as a potential cutoff for risk stratification. Furthermore, sacral malformations were strongly associated with NBD among patients with ARMs. HWR measurement could therefore be integrated into early diagnostic evaluation to facilitate timely multidisciplinary management.

## Introduction

Anorectal malformations (ARMs), particularly the high and intermediate types, are well known to be associated with genitourinary malformations, with some patients developing neurogenic bladder dysfunction (NBD). The reported prevalence of NBD among ARM patients ranges from 20% to 40% [1-3]. The underlying pathophysiology is thought to involve impaired sacral nerve development and spinal cord malformations, which disrupt detrusor-sphincter coordination [3]. As a result, patients with ARMs complicated by NBD often require lifelong clean intermittent catheterization (CIC) to preserve renal function and prevent urinary tract deterioration [4]. While fecal incontinence has been widely recognized as a major complication of ARMs, NBD remains relatively underexplored. NBD can severely impair quality of life, as affected patients require lifelong bladder management and face risks of recurrent urinary tract infections, renal dysfunction, and social limitations due to catheterization dependence [4]. Several imaging studies have attempted to predict bladder dysfunction based on spinal and pelvic morphology [5], yet few have evaluated early postnatal bladder configuration as a noninvasive predictive marker.

Recently, Kumano et al. [6] reported that the bladder height-to-width ratio (HWR), which has been used to assess bladder deformity and is important for diagnosing NBD [7], was correlated with high bladder pressure in patients with myelomeningocele. The current study, therefore, retrospectively evaluated the HWR among patients who had ARMs with NBD, focusing particularly on neonates, to determine its clinical relevance and predictive potential.

## Materials and methods

**Figure 1:**
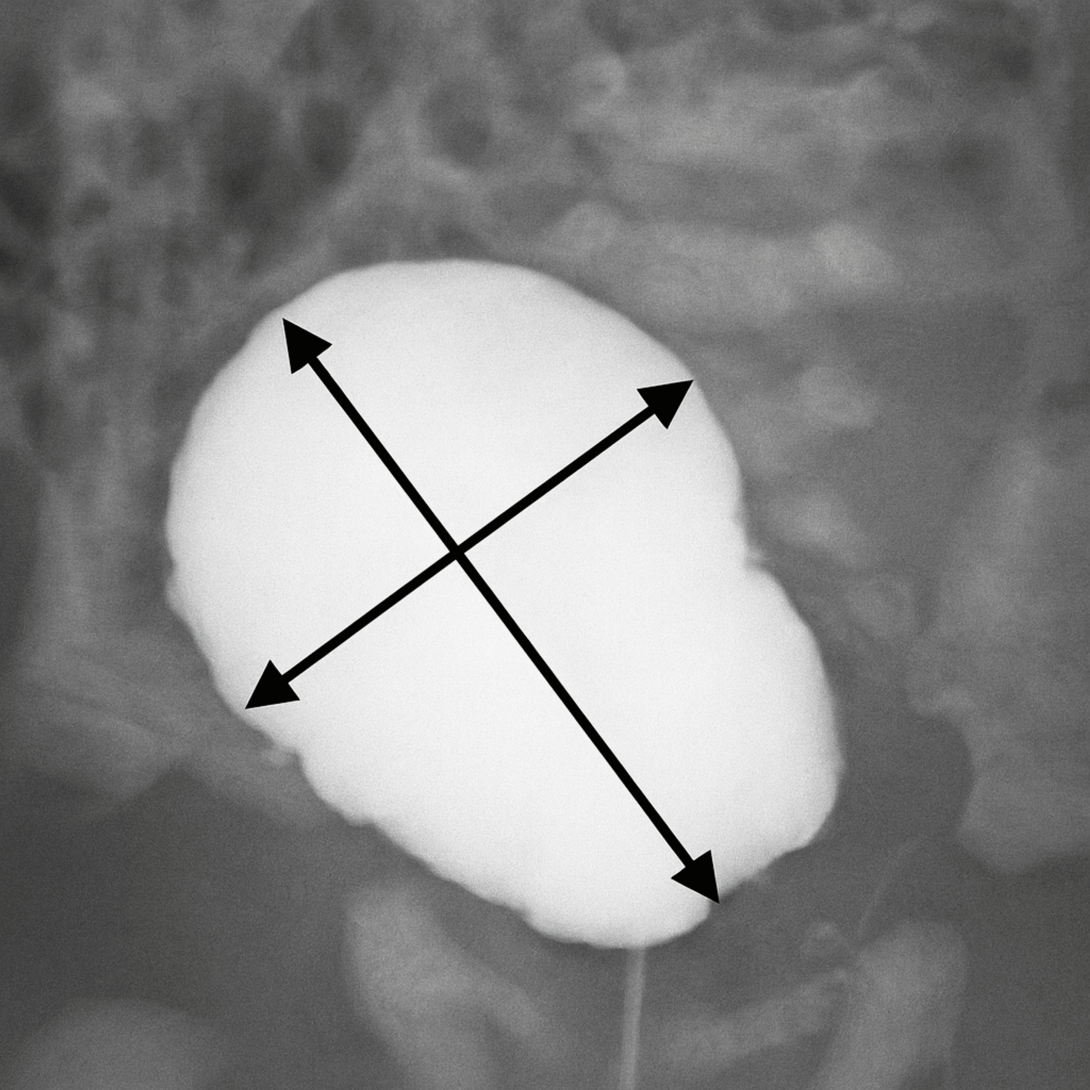
Cystography upon initial diagnosis of ARMs (cropped and enhanced). We measured and calculated the height-to-width ratio (maximum height/maximum width). This patient’s cystographic findings already showed bladder deformity. ARMS, anorectal malformations.

We herein investigated the relationship between HWR and NBD among patients with ARMs who underwent surgery at our hospital’s Department of Pediatric Surgery from January 2000 to December 2018. Among the 52 patients who underwent surgery, 48 underwent laparoscopic-assisted anorectoplasty (LAARP), while four underwent posterior sagittal anorectoplasty (PSARP). The PSARP group was subsequently excluded, given its small sample size, and to eliminate the effects of different procedures. Two pediatric urologists measured the HWR via cystography using identical measurement criteria to diagnose ARMs and determine their type before surgery. Accordingly, 33 (68.7%) and 15 (31.2%) patients were identified to have high and intermediate ARMs. Cystography was performed by a pediatric surgeon at our hospital within 24 h after birth using a 6-Fr catheter (Figure 1). Although the timing of cystography was consistent across the cohort, minor variations in bladder filling volume could not be completely standardized. Such variability may have affected the measured HWR values to a limited extent. Nevertheless, all examinations were performed under comparable neonatal conditions, minimizing systematic bias. The 48 patients who underwent LAARP were then divided into two groups: the NBD group (eight males and no female patients), who needed CIC, and the non-NBD group (36 males and four females), who needed no CIC. Using cystography results, the bladder’s maximum height and maximum width were calculated and compared between the NBD and non-NBD groups. Odds ratios (ORs) for spinal abnormalities, sacral malformations, and genetic abnormalities were also examined in both groups. Plain abdominal radiography and spinal magnetic resonance imaging were used to determine spinal abnormalities and sacral malformations. 　

Statistical analyses

Initially, the F test was used to determine whether the variance in the HWR was similar in the NBD and non-NBD groups (p = 0.309). Thereafter, the Kolmogorov-Smirnov test was used to determine the normality of data distribution in each group (NBD group: p = 0.968, non-NBD group: p = 0.780). Accordingly, the Student t-test was used to compare whether significant differences in the HWR existed between the groups. Receiver operating characteristic (ROC) curves were then constructed using the aforementioned findings to calculate the sensitivity and specificity of HWR. The areas under the ROC curve (AUC) and 95% confidence intervals (CIs) were then calculated. Thereafter, the optimal cutoff value for predicting NBD was identified. Odds ratios (ORs) were calculated using Fisher’s exact test for categorical variables.

All statistical analyses were performed using EZR (Eazy R, version 1.54; Jichi Medical University, Tochigi, Japan), which is a freely available graphical user interface for R and is distributed under the GNU General Public License version 2 (GPL-2) [8].

This study was approved by the Saitama Children's Medical Center Ethics Committee (Approval No.: 2025-02-001). Due to its retrospective design and the use of anonymized data, the requirement for informed consent was waived. The study was conducted in accordance with the Declaration of Helsinki.

## Results

The mean age of the entire cohort was 11.54 years (range, 9.57-13.5 years), while that for the NBD and non-NBD group was 8.7 ± 6.2 years and 11.5 ± 6.7 years, respectively (p = 0.302, Mann-Whitney U test; Table 1).

**Table 1:** Comparison of patient backgrounds between the non-NBD and NBD groups. NBD, neurogenic bladder dysfunction; HWR, height-to-width ratio. Student’s t-test was used for continuous variables, Fisher’s exact test was used for categorical variables. Statistical analyses were performed using open-access software (EZR, Jichi Medical University), distributed under free academic-use licenses.

Variable	Total (n=48)	NBD group (n=8)	Non-NBD group (n=40)	t value	p value (Student's t-test)
Age (years) (SD)	11.56 (9.60–13.52)	7.88 (2.67–13.07)	12.28 (10.13–14.41)	-1.62	0.131
Sex (male/female)	44/4	8/0	36/4	-	1.00
HWR (95% CI)	1.517 (1.419-1.616)	1.942 (1.652-2.231)	1.432 (1.346-1.519)	4.33	0.0000295

**Figure 2:**
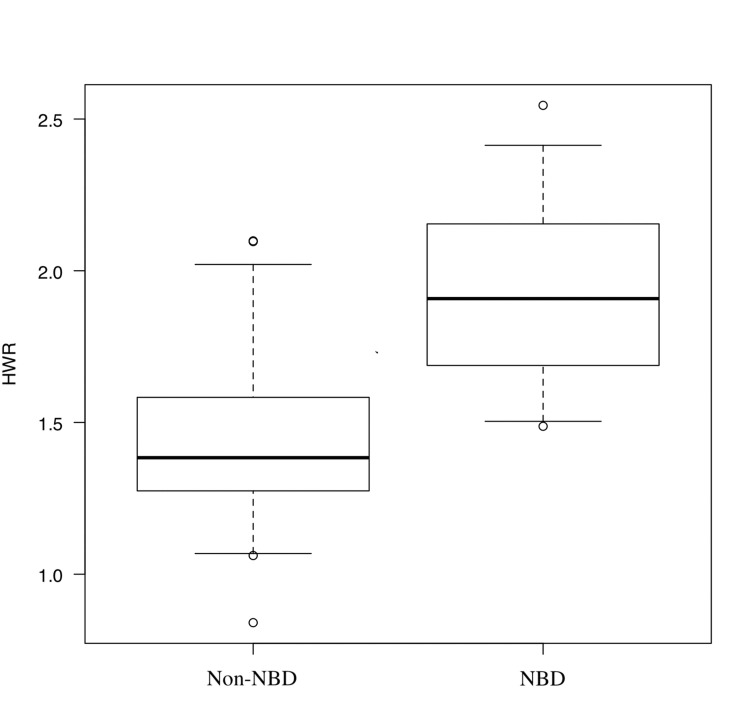
Comparison of HWR in non-NBD and NBD. The box plots show the median and interquartile range of the height-to-width (HWR) ratio. The mean HWR was 1.324 ± 0.270 in the non-NBD (neurogenic bladder dysfunction) group and 1.908 ± 0.346 in the NBD group (95% CI of the difference : 0.288–0.730; p = 0.000029, Student’s t-test). Statistical analyses were performed using open-access software (EZR, Jichi Medical University),  distributed under free academic-use licenses.

The NBD and non-NBD group had a median HWR of 1.942 (1.652-2.231) and 1.432 (1.346-1.519), respectively (Figure 2).

**Figure 3:**
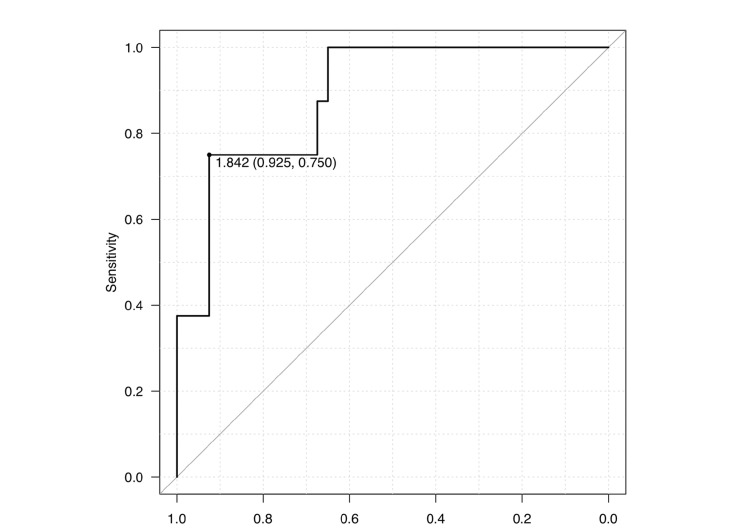
ROC curves for HWR in non-NBD and NBD groups. Receiver operating characteristic (ROC) curve showing the cutoff value of the height-to-width ratio (HWR) for differentiating between non-NBD (neurogenic bladder dysfunction) and NBD groups. The area under the curve (AUC) was 0.888 (95% CI: 0.773–1.000), indicating excellent discrimination between the two groups. Statistical analyses were performed using open-access software (EZR, Jichi Medical University),  distributed under free academic-use licenses.

The ROC curve for HWR revealed a cutoff value of 1.842 (AUC = 0.888, 95% CI, 0.773-1), with a sensitivity and specificity of 92.5% and 75%, respectively (Figure 3).

The ORs for spinal abnormality, sacral malformations, genetic abnormalities, and ARM type were 3.02 (95% CI: 0.502-22.385; p = 0.2358), of was 10.7686 (95% CI: 1.569-93.34; p = 0.00603), 1.8604 (95% CI: 0149-14.35; p = 1.8604), 3.6856 (95% CI: 0.404-181.64; p = 0.4056), respectively (Table 2).

**Table 2:** Comparison of risk factors between non-NBD and NBD groups. NBD, neurogenic bladder dysfunction; ARMs, anorectal malformations. Odds ratios (ORs) were calculated using Fisher’s exact test (two-tailed). "+" indicates presence, and "-" indicates absence of each anomaly. Statistical analyses were performed using open-access software (EZR, Jichi Medical University),  distributed under free academic-use licenses.

Variable	Categry	NBD group	Non-NBD group	Odds ratio (95% CI)	p value (Fisher’s exact test)
Spinal abnormalities	+/-	5/3	14/26	3.02 (0.50-22.39)	0.236
Sacral malformations	+/-	5/3	5/35	10.77(1.57-93.34)	0.006
Genetic abnormalities	+/-	2/6	6/34	1.86 (0.15-14.35)	0.605
Type of ARMs	High/ Intermediate	7/1	26/14	3.69 (0.40-181.64)	0.406

## Discussion

The results of this study suggest that patients with a high HWR (greater than 1.842) on cystography at the time of ARM diagnosis are significantly more likely to develop neurogenic bladder dysfunction (NBD) and subsequently require clean intermittent catheterization (CIC). In particular, patients with ARMs who had sacral malformations were found to have a higher risk for NBD compared to those with other associated anomalies. The simplicity and objectivity of HWR measurement make it an attractive screening tool, especially in facilities where urodynamic study (UDS) is not routinely available [4]. These findings collectively indicate that morphological bladder changes reflected by an increased HWR may serve as an early marker of neurogenic compromise secondary to sacral or spinal malformations.

The longitudinal elongation of the bladder has been attributed to the intrinsic architecture of the bladder wall. The bladder consists of three layers of smooth muscle: the outer and inner layers are oriented longitudinally, whereas the middle layer is circularly arranged [9]. Andersson et al. demonstrated that during bladder filling, smooth muscle cells relax, elongate, and rearrange within the wall, with elevated intravesical pressure resulting in longitudinal elongation of the bladder body [10]. The HWR has therefore been utilized as a quantitative indicator of such structural deformation, with a higher HWR representing elevated mean intravesical pressure.

Kumano et al. reported that the HWR serves as a useful screening parameter for detecting high bladder pressure in patients with myelomeningocele [6]. Interestingly, their proposed cutoff value (1.40) was lower than that identified in the present study (1.842). From an embryologic standpoint, myelomeningocele develops by gestational weeks 4-6, whereas ARMs form between weeks 6-8. In myelomeningocele, bladder dysfunction gradually evolves after birth as progressive neural injury occurs due to gravitational strain on the spinal cord. In contrast, ARMs may involve bladder dysfunction originating in utero, with deformation present at birth. This hypothesis, although speculative, highlights the potential developmental origin of NBD in ARMs and warrants further study.

UDS remains the gold standard for assessing bladder compliance, capacity, and detrusor pressures [10]; however, it presents significant challenges in neonates because of poor cooperation and variable abdominal pressure during crying [11]. Moreover, UDS requires specialized equipment and expertise not available in all institutions, whereas HWR measurement is simple, reproducible, and inexpensive. Approximately 30% of male patients with high ARMs have been reported to exhibit abnormal UDS findings [12]. Another study demonstrated that ARM patients with recto-bladder neck fistulas exhibited a median leak point pressure of 56 cmH₂O, with 75% showing detrusor overactivity [12]. These studies, however, involved non-neonatal populations, limiting generalizability to early infancy.

Borg et al. classified NBD in ARMs into two patterns: group A, characterized by detrusor overactivity with early leakage during bladder filling, and group B, characterized by increased capacity and incomplete bladder emptying [3]. They observed that most boys fell into group A, failing to store urine, while most girls belonged to group B. Both increased bladder pressure in group A and high residual urine in group B can contribute to longitudinal bladder elongation. Since HWR measurement alone cannot distinguish between these physiological subtypes, a combination of UDS and cystography may provide complementary diagnostic insight.　

In the present study, all patients with neurogenic bladder dysfunction were male.

Borg et al. reported that the risk of bladder dysfunction in ARM patients was primarily associated with spinal cord malformation and ARM severity, rather than sex [3].

Therefore, the all-male composition of our NBD group may reflect sampling bias related to the small cohort size and male predominance of ARM cases.

Our study demonstrated that approximately 16.7% of patients with ARMs developed NBD. This result is consistent with previous reports, which described an incidence of approximately 25% [13] to as high as 50% [10]. Pediatric surgeons and urologists should therefore remain vigilant regarding the elevated risk of NBD among patients with ARMs and ensure early communication and collaborative management to prevent functional deterioration.

The relationship between ARMs and urologic dysfunction remains multifactorial, influenced by the wide spectrum of associated anomalies such as spinal defects and sacral malformations. Even patients with similar anatomic diagnoses may exhibit different clinical courses, complicating management decisions. In this study, sacral malformations were the strongest independent risk factor for NBD, consistent with prior reports [10,13]. Among these, sacral agenesis appears to play a particularly decisive role in the development of NBD. Therefore, patients with ARMs and sacral anomalies should undergo close, long-term multidisciplinary monitoring [14].

Long-term outcomes are not trivial: one study reported that approximately 1% of 321 ARM patients progressed to end-stage renal disease requiring kidney transplantation [15]. NBD contributes to vesicoureteral reflux, hydronephrosis, and recurrent urinary tract infections, often leading to prolonged hospitalization and reduced quality of life. Consequently, long-term follow-up by both pediatric surgeons and urologists is essential to preserve renal function [13]. HWR should be evaluated at the time of ARM diagnosis via cystography, and patients with values exceeding 1.842 should be closely followed for early signs of NBD. Furthermore, pediatric urologists should periodically assess bladder morphology using ultrasonography and renal function via laboratory testing, irrespective of the presence of early urological events [10]. Pharmacologic management with anticholinergic agents may be beneficial in selected at-risk patients, though such medications can be challenging in those with fecal incontinence due to gastrointestinal side effects. Moreover, Wood and Levitt highlighted that renal and bladder sequelae may persist into adulthood, emphasizing the need for continuous multidisciplinary follow-up [16]. Similarly, Chong et al. demonstrated that even decades after definitive repair, ARM patients remain at risk for progressive urinary tract deterioration, reinforcing the necessity of lifelong urological surveillance [17].

Several limitations of this study should be acknowledged. First, this was a single-institution study with a limited sample size. The confidence intervals of the odds ratios were relatively wide in this study. This may be attributed to the small sample size and the limited number of NBD events, which reduced statistical precision. Therefore, the results should be interpreted with caution, and further validation in a larger cohort is warranted. Second, potential bias due to variation in surgical techniques could not be entirely excluded. Third, although this study included patients aged over two years, symptoms requiring CIC may develop at different ages. In addition, potential measurement bias should be acknowledged because the study was based on retrospective imaging review, and bladder filling was not standardized. Minor variations in bladder volume at the time of cystography could have affected the HWR measurements.

To the best of our knowledge, this is the first study to evaluate the association between ARMs and NBD using HWR as a morphological indicator. Future multicenter, prospective studies with larger cohorts to determine whether HWR can be integrated into standardized neonatal ARM evaluation protocols, and longer follow-up are warranted to validate our findings, determine optimal cutoff values, and further clarify the clinical utility of HWR as a non-invasive predictive tool for early detection of bladder dysfunction in ARM patients.

## Conclusions

This study demonstrates that neonates with high and intermediate anorectal malformations (ARMs) who exhibit a bladder height-to-width ratio (HWR) greater than 1.842 are at a significantly higher risk of developing neurogenic bladder dysfunction (NBD). The HWR provides a simple, objective, and non-invasive parameter that can assist clinicians in identifying high-risk patients even before urodynamic testing becomes feasible. Given its ease of measurement and reproducibility, HWR can be readily incorporated into routine diagnostic cystography, providing an early opportunity for risk stratification and individualized management planning.

In addition, the strong association between sacral malformations and NBD reinforces the necessity of early and multidisciplinary follow-up involving pediatric surgeons, urologists, and radiologists. Implementing early monitoring protocols (including renal function assessment, ultrasonography, and, when appropriate, pharmacologic intervention) may help prevent irreversible upper urinary tract deterioration. From a broader clinical perspective, integrating HWR assessment into the initial diagnostic workup for ARMs may not only standardize care pathways but also support early parental counseling regarding the potential for bladder dysfunction. Future multicenter, prospective studies with larger sample sizes are warranted to validate these findings, define standardized cutoff values, and determine whether early intervention guided by HWR screening can improve long-term renal and continence outcomes.
